# European mitochondrial haplogroups predict liver-related outcomes in patients coinfected with HIV and HCV: a retrospective study

**DOI:** 10.1186/s12967-019-1997-x

**Published:** 2019-07-26

**Authors:** Teresa Aldámiz-Echevarría, Salvador Resino, José M. Bellón, María A. Jiménez-Sousa, Pilar Miralles, Luz M. Medrano, Ana Carrero, Cristina Díez, Leire Pérez-Latorre, Chiara Fanciulli, Pilar Garcia-Broncano, Juan Berenguer

**Affiliations:** 1 Unidad de enfermedades infecciosas/VIH, Hospital General Universitario Gregorio Marañón, Madrid, Spain; 2 Fundación para la Investigación Biomédica, Instituto de Investigación Sanitaria Gregorio Marañón (IiSGM), Madrid, Spain; 3 Unidad de Infección Viral e Inmunidad, Centro Nacional de Microbiología, Instituto de Salud Carlos III, Carretera Majadahonda-Pozuelo, Km 2.2, 28220 Majadahonda, Madrid, Spain

**Keywords:** Mitochondria, mtDNA haplogroups, HIV, Chronic hepatitis C, Cirrhosis, Liver-related outcomes

## Abstract

**Background:**

Mitochondrial DNA (mtDNA) haplogroups have been associated with advanced liver fibrosis and cirrhosis in patients coinfected with human immunodeficiency virus (HIV) and hepatitis C virus (HCV). Our aim was to determine whether mtDNA haplogroups are associated with liver-related events (LREs) in HIV/HCV-coinfected patients.

**Methods:**

We carried out a retrospective cohort study in HIV/HCV-coinfected patients who were potential candidates for therapy with interferon and ribavirin (IFN/Rib) between 2000 and 2009. The primary endpoint was the occurrence of LREs (decompensation or hepatocellular carcinoma). mtDNA genotyping was performed using the Sequenom MassARRAY platform. We used Fine and Gray proportional hazards model to test the association between mtDNA haplogroups and LREs, considering death as a competitive risk.

**Results:**

The study population comprised 243 patients, of whom 40 had advanced fibrosis or cirrhosis. After a median follow-up of 7.7 years, 90 patients treated with IFN/Rib achieved sustained viral response (SVR), 18 patients had LREs, and 11 patients died. Patients with haplogroup H had lower cumulative incidence than patients with other haplogroups (*p *= 0.012). However, patients with haplogroup T had higher cumulative incidence than patients with other haplogroups (*p *= 0.074). In the multivariate analysis, haplogroup T was associated with an increased hazard of developing LREs [adjusted subhazard ratio (aSHR) = 3.56 (95% CI 1.13;11.30); *p *= 0.030]; whereas haplogroup H was not associated with lower hazard of LREs [aSHR = 0.36 (95% CI 0.10;1.25); *p *= 0.105]. When we excluded patients who achieved SVR during follow-up, we obtained similar SHR values.

**Conclusions:**

European mitochondrial haplogroups may influence the natural history of chronic hepatitis C.

**Electronic supplementary material:**

The online version of this article (10.1186/s12967-019-1997-x) contains supplementary material, which is available to authorized users.

## Background

Hepatitis C virus (HCV) infection is the leading cause of end-stage liver disease, hepatocellular carcinoma (HCC), and liver-related death in developed countries [1]. It is estimated that about 10–20% individuals with chronic hepatitis C develop cirrhosis around 20 to 30 years after acquiring HCV and that those who develop cirrhosis have a 1% to 5% annual risk of developing HCC and a 3% to 6% annual risk of liver decompensation [2, 3]. However, chronic hepatitis C is highly variable among individuals, ranging from minimal histological changes to extensive fibrosis and cirrhosis [3].

Although we lack predictive models to estimate the risk of fibrosis and clinical progression in individuals early on in their HCV infection, several factors are associated with increased risk of progression of liver fibrosis, including age, male gender, high alcohol intake, obesity, insulin resistance, type 2 diabetes, coinfection with human immunodeficiency virus (HIV) hepatitis B virus, and immunosuppressive therapy [3]. Host nuclear genetic factors also influence the natural history of HCV infection and include viral clearance, progression of fibrosis, and development of cirrhosis and HCC [3, 4].

In the last decade, the role of mitochondrial genetics in human disease has been increasingly recognized. Mitochondria provide energy to eukaryotic cells via oxidative phosphorylation and regulate cellular survival via control of apoptosis [5]. Mutations in mitochondrial DNA (mtDNA) have been acquired throughout history by natural selection owing to adaptation to environmental conditions [6]. Consequently, the human population can be subdivided into several mitochondrial clades or haplogroups, which are defined based on specific mtDNA polymorphisms [7]. In Europe, macro-haplogroups HV, U, and JT are about 90% of the population [8]. Of them, 50% of the Europeans belongs to the macro-haplogroup HV and 45% are haplogroup H. The macro-haplogroups U is divided into many sub-haplogroups that comprise approximately 20% of the Caucasian population. The macro-haplogroup JT is subdivided in haplogroups J (8% of the population) and T (9% of Europeans).

MtDNA haplogroups have been increasingly recognized as contributors to diseases such as cancer, sepsis, diabetes, and degenerative diseases [9, 10]. However, there is now clear evidence that mtDNA variants within haplogroups may be the trigger of the large number of diseases with which mtDNA haplogroups have been linked. Functional studies are scarce and technically complicated because the likely biochemical effect of mtDNA polymorphisms may be subtle. In addition, many of these mtDNA polymorphisms are found in genomes that contain other polymorphisms, which may be interacting [11]. Cybrid technology is widely used for the study of phenotypical effect of mutations in the mtDNA. In this “in vitro” model, it has been observed that haplogroup H cybrids contain higher levels of mtDNA and mRNA, growing faster, have a higher membrane potential, and consume more oxygen than haplogroup Uk and T cybrids. Other studies have reported that haplogroup J cybrids have slower rate of assembly of the mitochondrial complexes and lower ATP and ROS production than haplogroup H cybrids [11]. In addition, some of these findings have also been described in patients, although the article number is smaller.

Preliminary studies have also shown associations between mtDNA haplogroups and clinical outcomes in patients with HIV infection [12, 13], including clinical progression [14–17], CD4+ T cell recovery after combination antiretroviral therapy (cART) [18], metabolic disorders [19, 20], and toxicities due to nucleoside reverse-transcriptase inhibitors (e.g., peripheral neuropathy and lipoatrophy) [21, 22]. In previous reports, an association between major European mtDNA haplogroups and liver fibrosis in HIV/HCV-coinfected patients were also found [23, 24], but the design of this studies was cross-sectional.

### Objective

In the current study, we aimed to determine whether mtDNA haplogroups are associated with clinical outcomes, including liver-related events (LREs) and death, in HIV/HCV-coinfected patients through a longitudinal study.

## Materials and methods

### Study population

We carried out retrospective study in a cohort of 243 HIV/HCV-coinfected patients who had been evaluated for interferon and ribavirin therapy at Hospital Gregorio Marañón (Madrid, Spain) between 2000 and 2009. The patients were negative for hepatitis B surface antigen, and a DNA sample was available for each one. The selection criteria for anti-HCV therapy at the time were detectable HCV RNA by polymerase chain reaction (PCR), no clinical evidence of hepatic decompensation, CD4+ lymphocyte count higher than 200 cells/µL, and stable cART for at least 6 months or no need for cART according to the guidelines used during the study period. Patients with active opportunistic infections and severe concurrent medical conditions (e.g., poorly controlled hypertension, heart failure, poorly controlled diabetes mellitus, and severely reduced renal function) were excluded. A period of at least 6 months of abstinence from heroin and cocaine was also required in patients with a history of injection drug use. In addition, 162 healthy blood donors (negative for HIV, HCV, and hepatitis B virus infection) from the “Centro de Transfusión de la Comunidad de Madrid” participated as a control group. Their age and gender matched those of the HIV-infected patients.

The study was conducted in accordance with the Declaration of Helsinki and patients gave their informed consent for the study. The Institutional Review Board and the Research Ethic Committee of the Instituto de Salud Carlos III approved the study (# CEI PI 41_2014). Patients included in this study signed a written informed consent.

### Clinical and laboratory data

Baseline clinical and epidemiological data were recorded the day the liver biopsy was performed or the day the patients were evaluated for interferon and ribavirin therapy if a liver biopsy was not performed. We considered intake of > 50 g of alcohol per day for ≥ 12 months as high. A blood sample was taken from each patient before liver biopsy for a complete blood count, coagulation testing, liver panel, basic metabolic panel, CD4+ T-cell count, plasma HIV-RNA, and plasma HCV-RNA. In addition, a serum sample was immediately frozen and stored at − 70 °C for further assays.

Outpatient percutaneous liver biopsies were performed routinely by PM and JB following widely accepted recommendations to assess whether patients were candidates for therapy with interferon and ribavirin [25]. Liver biopsy samples were examined by two pathologists who agreed on the scoring of fibrosis following the criteria of the METAVIR Cooperative Study Group [26], as follows: F0, no fibrosis; F1, portal fibrosis; F2, periportal fibrosis or rare portal–portal septa; F3, fibrous septa with architectural distortion and no obvious cirrhosis (bridging fibrosis); and F4, cirrhosis. Fibrosis was also staged at baseline using the FIB-4 index, as follows: [age (years) × aspartate aminotransferase (AST) (U/L)]/[platelet count (10^9^/L) × alanine aminotransferase (ALT) (U/L)^1/2^] [27]. In this study, advanced fibrosis was defined as a METAVIR stage ≥ F3 or a FIB-4 value ≥ 3.25.

Follow-up information included treatment of HCV infection and response, LREs, and mortality. This information was recorded retrospectively from hospital clinical records. Sustained virologic response (SVR) was defined as undetectable serum HCV-RNA level 24 weeks after discontinuation of interferon and ribavirin. The LREs analyzed included ascites, hepatic encephalopathy, variceal bleeding, and HCC. Paracentesis or ultrasound confirmed ascites. Hepatic encephalopathy was established based on clinical findings, laboratory parameters, and neuroimaging techniques; after the reasonable exclusion of HIV-associated encephalopathy. The gastroesophageal bleeding was confirmed by endoscopy whenever possible. Diagnosis of HCC was based on noninvasive imaging tests or pathology findings [28]. The administrative censoring date was June 30, 2013.

### mtDNA genotyping

Total DNA was extracted from peripheral blood using Qiagen columns (QIAamp DNA Blood Midi/Maxi, Qiagen, Hilden, Germany). DNA samples were genotyped using the MassARRAY platform (Sequenom, San Diego, CA, USA) based on the iPLEX^®^ Gold assay design. All individuals were classified within the European macrocluster of N and further separated into the most common haplogroups or major groups (HV, IWX, U, and JT) and haplogroups (H, V, pre-V, J, T, I, W and X) according to 14 polymorphisms in the mtDNA (see Additional file 1: Figure S1), as previously described [18]. All patients were of European ancestry because individuals not within the N macro-cluster were excluded from the study.

### Outcome variables

The primary endpoint was the occurrence of LREs. This endpoint was chosen because it is the most appropriate outcome in patients with the compensated liver disease, whereas death is the most relevant outcome in those with the decompensated liver disease [29]. For patients who had more than one liver-related event, only the first was included in the analysis.

### Statistical analysis

All analyses were performed using Stata software (version 14.0; Stata Corporation, College Station, TX, USA). All p-values < 0.05 were considered significant.

We used the Fine and Gray proportional hazards model (Stata’s stcrreg module) to test the association between mtDNA haplogroups and outcomes, considering non-liver death as a competitive risk [30], in separate individual models (each haplogroup separately). SVR was analyzed as a time-dependent covariate. Additionally, the analysis was also performed without patients who achieved SVR. This test gives the SubHazard Ratio (SHR) with 95% confidence intervals (95% CI) as a measure of risk [31]. The regression tests were adjusted for the most significant covariates associated with each of the outcome variables (p < 0.05), and over-fitting of the regression was avoided. The covariates used initially were gender, age, injection drug use, high alcohol intake, AIDS diagnosis, nadir CD4+, HCV genotype, HCV viral load, FIB-4, cART, HCV antiviral therapy, and SVR. We adjusted for FIB-4 instead of biopsy stage (METAVIR) because FIB-4 has been shown to outperform liver biopsy in the assessment of prognosis (LREs and death) in HIV/HCV-coinfected patients [32]. Additionally, we used the Stata’s stcompet and stpepemori implements to generate cumulative incidence in the presence of competing events and p-values, respectively [30].

## Results

### Characteristics of the study population

A total of 243 HIV/HCV-coinfected patients who self-identified as “white” and had a western European, or N, mitochondrial macro-cluster, were included in the analysis. The demographic and clinical characteristics of the patients are summarized in Table 1. In brief, 74.9% were male, the median age was 40.7 years, 86% acquired HIV by injection drug use, 30.2% had prior AIDS-defining conditions, 81.5% were on cART, 12.3% reported a high intake of alcohol, the median baseline CD4+ T-cell count was 485 cells/mm^3^, 74.2% had an undetectable HIV viral load, 76.9% were infected by genotypes 1 or 4, and 78.4% had HCV RNA ≥ 500,000 IU/mL. A total of 19% patients had advanced fibrosis or cirrhosis.

**Table 1:** Clinical and demographic characteristics of the study cohort

Demographic/clinical variable	All patients	Excluding patients with SVR
No.	243	153
Male sex	182 (74.9%)	118 (77.1%)
Age—year	40.7 (37.7–44.8)	41.6 (38.2–45)
Prior injection drug use	209 (86%)	132 (86.3%)
Current alcohol intake > 50 g/d	30 (12.3%)	21 (13.7%)
CDC category C	73 (30.2%)	56 (36.8%)
CD4+ T cells nadir—n/mm^3^	189 (77–306)	169 (59–273)
cART	198 (81.5%)	122 (79.7%)
Undetectable HIV viral load	178 (74.2%)	110 (73.3%)
Baseline CD4+ T cells—n/mm^3^	485 (346–679)	468 (340–667)
HCV genotype		
1.4	180 (76.9%)	129 (87.8%)
2.3	54 (23.1%)	18 (12.2%)
HCV-RNA ≥ 500,000 IU/mL	174 (78.4%)	113 (81.5%)
METAVIR fibrosis stage (n = 210)		
F0 or F1	109 (45.9%)	77 (50.3%)
F2	61 (25.1%)	35 (22.9%)
F3	20 (8.2%)	11 (7.2%)
F4	21 (8.6%)	11 (7.2%)
N/A	32 (13.2%)	19 (12.4%)
FIB-4 − median (IQR)	1.46 (1.03–2.06)	1.43 (1.08–1.92)
FIB-4 ≥ 3.25	24 (9.9%)	14 (9.2%)

Values are expressed as median (IQR) and absolute count (percentage)

*HCV* hepatitis C virus, *HIV-1* human immunodeficiency virus type 1, *HIV-RNA* plasma HIV load, *cART* combination antiretroviral therapy, *CDC* Centers for Disease Control and Prevention, *SVR* sustained virologic response

Additionally, Table 1 also shows the characteristics of the patients without those who achieved SVR.

### Liver-related outcomes

The median follow-up was 93 months (7.7 years). During that period, 18 patients had LREs, including ascites (n = 7), ascites plus HCC (n = 3), HCC (n = 3), ascites plus variceal bleeding (n = 1), ascites plus variceal bleeding plus HCC (n = 1), ascites plus spontaneous bacterial peritonitis plus variceal bleeding (n = 1), hepatic encephalopathy (n = 1), and hepatic encephalopathy plus HCC (n = 1). Liver fibrosis stage at baseline in these 18 patients was as follows: F4, n = 8; F3, n = 4; and F0 to F2, n = 6. The time to LREs during follow-up were 5.46 years (95% CI 2.94; 6.68) for all patients, 4.21 years (95% CI 1.48; 5.70) for cirrhotic patients, 5.77 years (95% CI 3.51; 7.43) for non-cirrhotic patients.

A total of 11 patients died during follow-up. The causes of death were liver-related death (n = 6), non–liver-related non–AIDS-related death (n = 4), and AIDS-related death (n = 1). At baseline, liver fibrosis was staged at F4 for 5 patients, F3 for 1 patient, and F0 to F2 for 5 patients. Non-liver-related non-AIDS-related death included non-AIDS-related bacterial infections (n = 2) and lung cancer (n = 2). The 2 patients who died from non–AIDS-related bacterial infections had F4 fibrosis at baseline.

During follow-up, 175 patients were treated with pegylated interferon plus ribavirin; of these, 90 achieved SVR. The LREs occurred in 15/153 (9.8%) patients without SVR and in 3/90 (3.3%) patients with SVR (p = 0.077). Liver fibrosis stage at baseline in these 15 patients was as follows: F4, n = 7; F3, n = 3; and F0 to F2, n = 5. The time to LREs during follow-up were 5.45 years (95% CI 2.94; 6.08) for all patients, 2.94 years (95% CI 1.01; 5.70) for cirrhotic patients, 5.77 years (95% CI 4.17; 7.05) for non-cirrhotic patients.

### European haplogroups and liver-related events

**Fig. 1:**
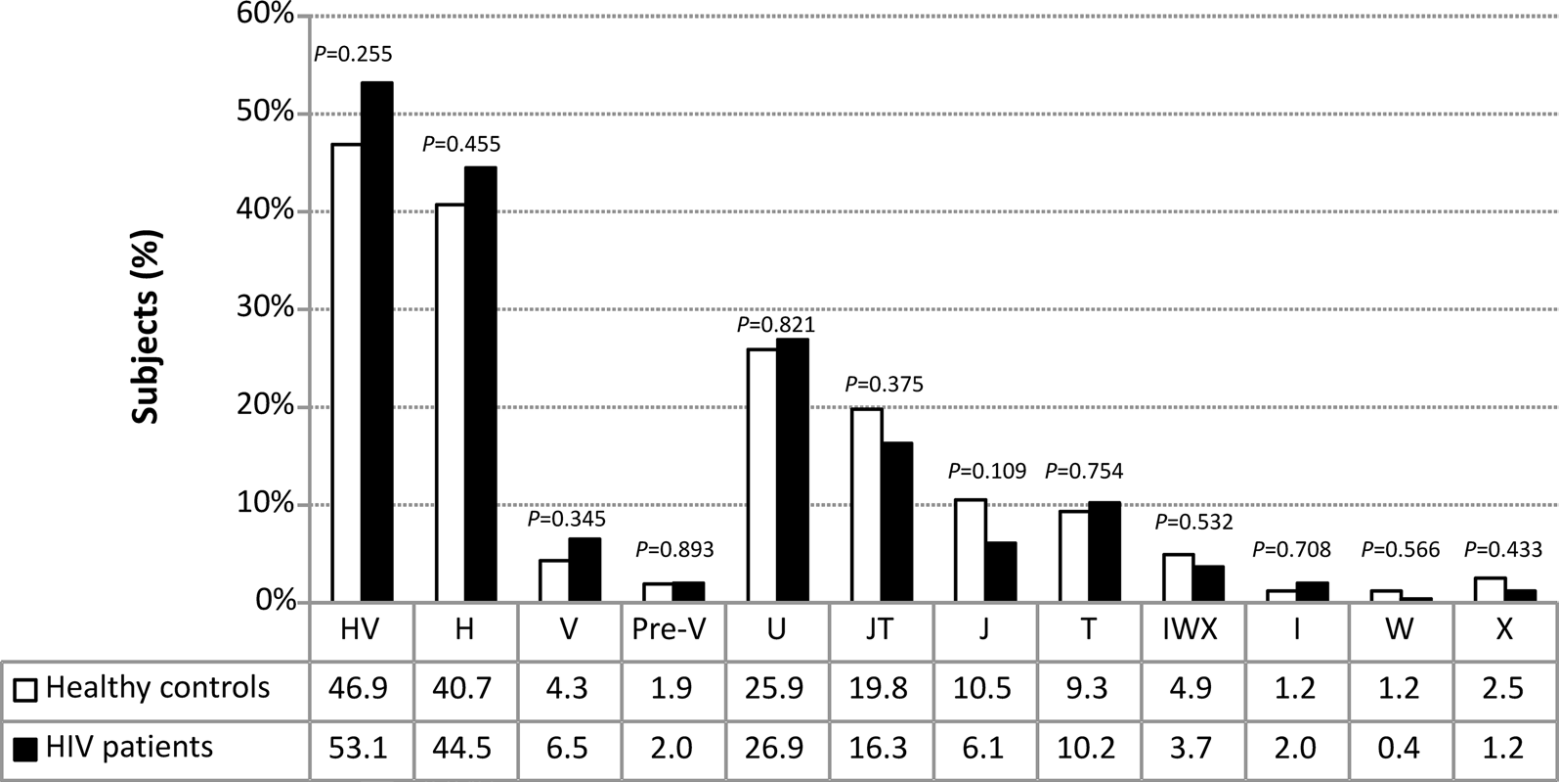
Frequencies of mtDNA haplogroups in 162 healthy controls and 245 HIV/HCV-coinfected patients

We did not find significant differences in the frequencies of mtDNA haplogroups between HIV/HCV-coinfected patients and healthy controls (Fig. 1), and the distribution of mtDNA haplogroups across the HIV-infected patients was similar to that reported by other authors studying HIV infection in a Caucasian population [16, 20, 22]. In HIV-infected patients, the haplogroups Pre-V, IWX, I, X, and W had frequencies of less than 5% (Fig. 1) and were excluded from the association analysis to minimize type II errors in the statistical analyses. The genetic association tests were performed on haplogroups H, V, U, J, and T. However; haplogroup J did not have a viable value because in one of the cells of Table 2 × 2 there was a value of zero.

**Fig. 2:**
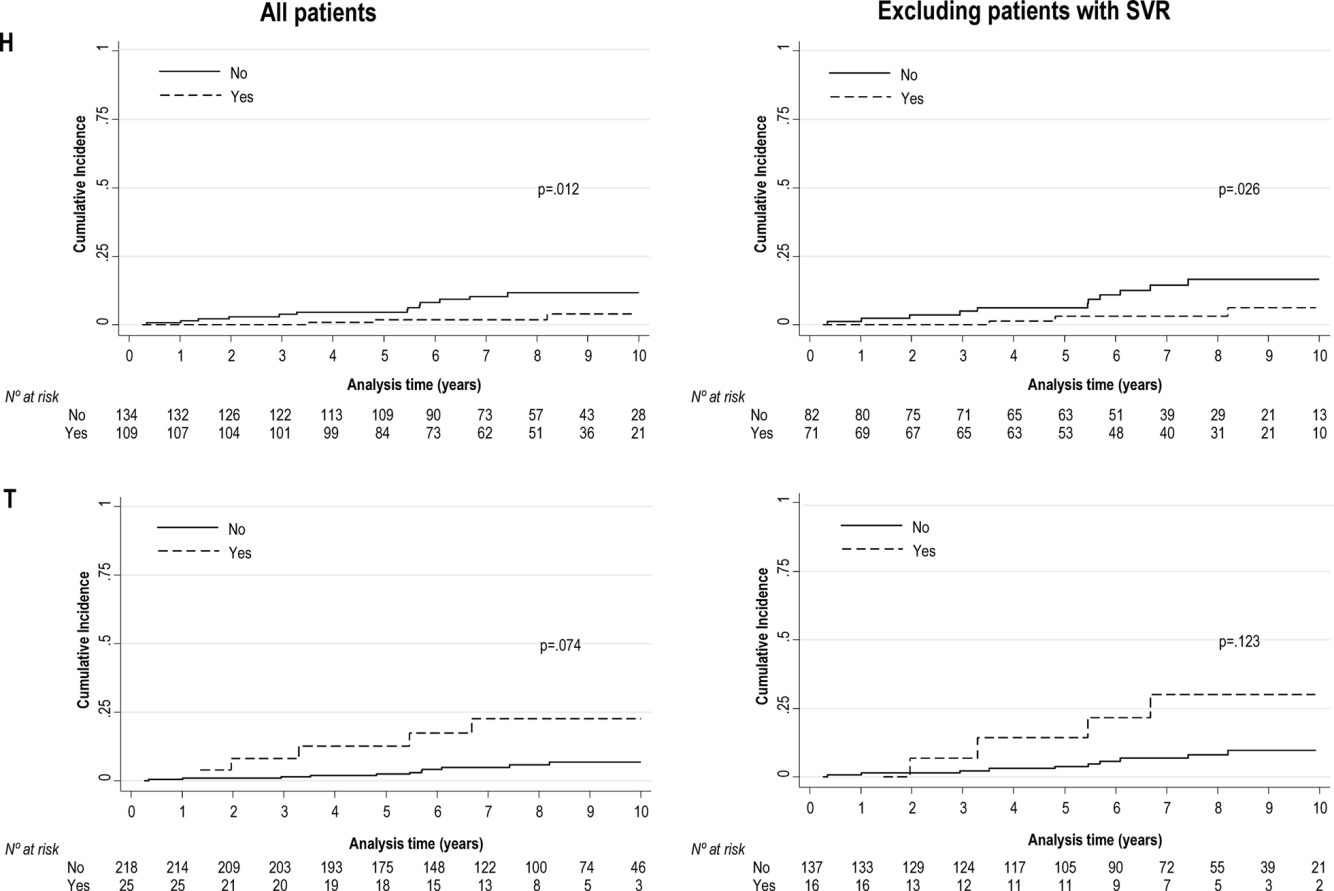
Cumulative incidence of liver-related events according to mtDNA haplogroups in HIV/HCV-coinfected patients

The frequencies of LREs according to the different haplogroups are shown in Table 2. The most outstanding cumulative incidence of LREs according to mtDNA haplogroups in HIV/HCV-coinfected patients are shown in Fig. 2. Patients with haplogroup H had lower cumulative incidence than patients with other haplogroups (*p *= 0.012), whereas patients with haplogroup T had higher cumulative incidence than patients with other haplogroups (*p *= 0.074). When patients with SVR during follow-up were excluded, we found patients with haplogroup H remained with lower cumulative incidence (*p *= 0.012) than patients with other haplogroups.

**Table 2:** Frequency of liver-related events in HIV/HCV-coinfected patients according to mitochondrial DNA haplogroup

mtDNA haplogroup	With haplogroup	All patients	Excluding patients with SVR
H	No	15/134 (11.2%)*	12/82 (14.6%)*
	Yes	3/109 (2.8%)	3/71 (4.2%)
V	No	17/227 (7.5%)	14/144 (9.7%)
	Yes	1/16 (6.3%)	1/9 (11.1%)
pre V	No	17/238 (7.1%)	14/149 (9.4%)
	Yes	1/5 (20%)	1/4 (25%)
HV	No	13/113 (11.5%)*	10/69 (14.5%)
	Yes	5/130 (3.8%)	5/84 (6%)
U	No	10/179 (5.6%)	9/115 (7.8%)
	Yes	8/64 (12.5%)	6/38 (15.8%)
J	No	18/228 (7.9%)	15/145 (10.3%)
	Yes	0/15 (0%)	0/8 (0%)
T	No	13/218 (6%)*	11/137 (8%)*
	Yes	5/25 (20%)	4/16 (25%)
JT	No	13/203 (6.4%)	11/129 (8.5%)
	Yes	5/40 (12.5%)	4/24 (16.7%)
I	No	18/238 (7.6%)	15/149 (10.1%)
	Yes	0/5 (0%)	0/4 (0%)
X	No	18/240 (7.5%)	15/151 (9.9%)
	Yes	0/3 (0%)	0/2 (0%)
W	No	18/242 (7.4%)	15/152 (9.9%)
	Yes	0/1 (0%)	0/1 (0%)
IXW	No	18/234 (7.7%)	15/146 (10.3%)
	Yes	0/9 (0%)	0/7 (0%)
	Total	18/243 (7.4%)	15/153 (9.8%)

*LRE* liver-related event, *SVR* sustained virological response

* p < 0.05

The results of the Fine and Gray competing-risks regression analysis for LREs in HIV/HCV-coinfected patients considering death as a competitive risk are shown in Table 3. The covariates selected for adjusting multivariate models were high alcohol intake, FIB-4, and SVR because had a significant association with LREs in univariate analysis (*p *< 0.05; data not shown). Thus, in the multivariate analysis, haplogroup T was associated with an increased hazard of developing LREs [adjusted SRH (aSHR) = 3.56 (95% CI 1.13; 11.30); *p *= 0.030]; whereas haplogroup H was not associated with lower hazard of LREs [aSHR = 0.36 (95% CI 0.10;1.25); *p *= 0.105]. When we excluded patients with SVR during follow-up, we obtained similar values. Haplogroup T remained associated with an increased hazard of developing LREs [aSHR = 4.26 (95% CI 1.15; 15.80); *p *= 0.030], and haplogroup H was not protected against the risk of LREs [aSHR = 0.44 (95% CI 0.12; 1.61); *p *= 0.214].

**Table 3:** Competing-risks regression analysis for liver-related events in HIV/HCV-coinfected patients considering death as a competitive risk

	All patients*		Excluding patients with SVR	
	aSHR (95% CI)	*p*	aSHR (95% CI)	*p*
Haplogroup H	0.36 (0.10; 1.25)	0.108	0.44 (0.12; 1.61)	0.214
Haplogroup V	0.20 (0.01; 3.95)	0.289	0.17 (0; 5.86)	0.325
Haplogroup J	NA	NA	NA	NA
Haplogroup T	3.56 (1.13; 11.3)	*0.030*	4.26 (1.15; 15.8)	*0.030*
Haplogroup U	1.81 (0.69; 4.76)	0.229	1.51 (0.48; 4.74)	0.480

*SVR* Sustained virologic response, *SHR* sub-hazard ratio, *aSHR* adjusted sub-hazard ratio (covariates selected: alcohol intake, FIB-4, and SVR*), *95% CI* 95% confidence interval, *NA* not available

* Sustained viral response was considered a time-dependent variable

## Discussion

In this article, with a cohort of HIV/HCV-coinfected patients who were followed up for a median of approximately 8 years, we found that the presence of mtDNA haplogroup T was related to an increased hazard of LREs. We also found that the presence of mtDNA haplogroup H was related to a reduced hazard of LREs in the competing-risks regression analysis, which did not achieve statistical significance in the adjusted regression analysis. To our knowledge, this is the first time mtDNA haplogroups have been found to be associated with LREs in HIV/HCV coinfected patients with chronic hepatitis C. Moreover, since there have been recently reported that the incidence of HCC has increased even after treatment of chronic hepatitis C [33–36], the results of this study also highlight the importance of mitochondrial genetics in patients with chronic hepatitis C in the future.

Several aspects of mitochondrial function are affected during HCV infection, such as the alteration of mitochondrial membrane potential, excessive production of reactive oxygen species (ROS), the significant fall in the level of adenosine triphosphate (ATP), and bioenergetic failure [37, 38]. Also, HCV-induced mitochondrial dysfunction may contribute to viral persistence by attenuating apoptosis of infected cells [39]. The mtDNA haplogroups tend to be associated with subtle differences in oxidative phosphorylation capacity and the generation of ROS [9]. Therefore, it is conceivable that the chronic oxidative stress due to HCV infection can be modulated by the DNA genetic. Of note, mtDNA haplogroup H has been associated with higher activity in the electron transport chain, leading to more significant quantities of ATP and ROS than other haplogroups, such as mtDNA haplogroup J and T, which exhibits lower energy efficiency [40, 41].

In a previous biopsy-based cross-sectional study of HIV/HCV-coinfected patients using the same cohort of patients [24], we found that mtDNA haplogroup H was strongly associated with reduced likelihood of having advanced fibrosis, cirrhosis, and fibrosis progression rates. In the present study, we have found haplogroup T was related to the development of LREs, whereas that haplogroup H was protective against LREs in Fine and Gray competing-risks regression analysis, but it did not achieve significant p-value in multivariate regression analysis. As discussed above, haplogroup T has a biochemical and energetic effect opposite to haplogroup H [40, 41]; and in our experience, we found opposing associations for other outcome variables, in other studies, when we compared haplogroup T versus haplogroup H in HIV infected subjects [18, 19].

The relevance of our findings for clinical practice can be limited by the fact that the data are from an era in which anti-HCV therapy was interferon-based rather that based on all oral direct-acting antivirals. However, our findings expand the knowledge about the pathogenesis of chronic hepatitis C in HIV-infected patients. Viewed from a broader perspective, our results suggest that mtDNA haplogroups may influence the natural history of chronic liver diseases of various etiologies; a notion that is supported by two recent studies. In the first study, the presence of mtDNA haplogroup L was found to exercise a protective effect against the development of non-alcoholic steatohepatitis and pericellular fibrosis in patients with non-alcoholic fatty liver disease [42]; in the second study, carried out in Chinese population with HCC, patients with haplogroup M8 had a lower survival rate than patients with haplogroup D4 [43]. The fact that a large proportion of the participants were treated with interferon could also limit the study’s implications on the natural history of HCV infection. However, we analyzed the SVR as a time-dependent covariate, and we also performed a sub-analysis discarding patients who achieved SVR, finding equivalent results in both cases. Non-responders to interferon therapy were kept in the study because this fact does not protect against the progression of chronic hepatitis C in the long term [44]. Another limitation is the small sample size, which may have impaired the ability to detect less robust associations, as we did not perform detailed analyses on some of the less common mtDNA haplogroups. Finally, our study was retrospective and consequently cannot prove causality. However, it must be considered that patients included in the study were highly selected and met a set of restrictive criteria for starting HCV treatment. Besides, they were followed in our institution by the same physicians throughout their disease, with clinical and laboratory assessment every 3 to 6 months according to standard practice with HIV-infected patients in Spain [45]. Furthermore, complications of cirrhosis were prevented or managed following protocols based on current clinical practice guidelines.

## Conclusions

In conclusion, our data suggest that mitochondrial haplogroups could influence the natural history of hepatitis C and may warrant further confirmatory longitudinal studies.

## Additional file

**Additional file 1.** List of European mitochondrial DNA (mtDNA) haplogroups with their defining mutation. Adapted of Hendrickson SL, Hutcheson HB, Ruiz-Pesini E, et al. Mitochondrial DNA haplogroups influence AIDS progression. AIDS 2008; 22:2429–2439.

## Data Availability

All data generated or analyzed during this study are included in this published article [and its additional information files].
