# Supplementary material for: European mitochondrial haplogroups predict liver-related outcomes in patients coinfected with HIV and HCV: a retrospective study
Source: J Transl Med. 2019 Jul 26;17:244. doi: 10.1186/s12967-019-1997-x (PMC6660654; doi:10.1186/s12967-019-1997-x)

**Figure S1.** List of European mitochondrial DNA (mtDNA) haplogroups with their defining mutation. Adapted of Hendrickson SL, Hutcheson HB, Ruiz-Pesini E, et al. Mitochondrial DNA haplogroups influence AIDS progression. AIDS 2008; 22:2429-2439.


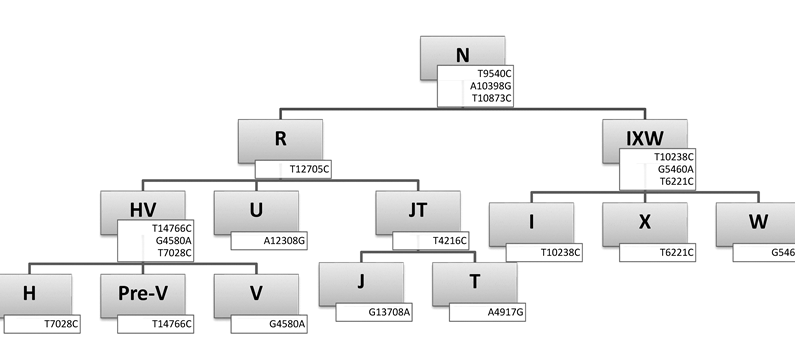

Supplement: Supplementary file 1 — Additional file 1. List of European mitochondrial DNA (mtDNA) haplogroups with their defining mutation. Adapted of Hendrickson SL, Hutcheson HB, Ruiz-Pesini E, et al. Mitochondrial DNA haplogroups influence AIDS progression. AIDS 2008; 22:2429–2439. [file 12967_2019_1997_MOESM1_ESM.docx]
